# Political cost, value balance, and the optimization of university public health emergency governance: an evolutionary game and small signal analysis

**DOI:** 10.3389/fpubh.2026.1824985

**Published:** 2026-09-16

**Authors:** Rui Wang, Haonan Wu, Yingyan Liu, Jianlin Li, Xin Li, Ruihao Li

**Affiliations:** 1 Tianjin University of Finance and Economics, School of Law, Tianjin, China; 2 Baotou Medical College, Inner Mongolia University of Science and Technology, Baotou, China; 3 Inner Mongolia Vocational College of Chemical Engineering, Hohhot, China; 4 Nankai University College of Tourism and Service Management, Tianjin, China; 5 Tianjin Ruiya Pharmacy Co., Ltd, Tianjin, China; 6 Tianjin Chest Hospital, Medical Insurance Department, Tianjin, China; 7 Nankai University Zhou Enlai School of Government, Tianjin, China; 8 Graduate School of Asia-Pacific Studies, Waseda University, Tokyo, Japan

**Keywords:** evolutionary game and small-signal model, optimization of governance system of universities, pandemic prevention and control, political cost, value balance

## Abstract

In major public-health emergencies, universities constitute core nodes within a multi-actor intersecting governance network composed of governments, universities, students, media and social-sector organizations. While uniform closed-campus management can maximize the protection of students' physical health, it frequently overlooks students' mental-health needs and value-recognition concerns, which may exacerbate tensions between universities and students. Taking political costs associated with university pandemic governance as the research subject, this study integrates systems theory and cost-benefit theory to construct a tripartite evolutionary-game model covering universities, social-media platforms and students. Evolutionarily stable strategies are derived by solving replicator-dynamic equations. A small-signal model is further introduced to analyze the stability, root-locus characteristics and participation factors of all eight equilibrium points. Python-based numerical simulation is also adopted to reveal the feedback effect of political costs on system stability. This paper presents a case analysis of responses and proactive adjustment outcomes of two universities during the 2022 Omicron wave. The findings are as follows: (1) University governance behavior serves as the dominant participation factor driving instability across different equilibrium states, whereas student (public) participation acts as the core stabilizing factor and the primary driver of political-cost consumption; (2) Under the optimal equilibrium, a moderate political-cost signal can both warn universities against exploiting information asymmetry and empower them to respond decisively to public-health crises, fostering a win-win tripartite synergy among universities, media and students; (3) When political costs are severely depleted, students will set aside economic considerations and prioritize health-related concerns by adopting negative behavioral responses, which destabilizes the governance system. On this basis, this paper puts forward a three-dimensional “system-process-value” framework. It optimizes the supply pathway of university pandemic-prevention policies through structural reorganization, process re-engineering and value allocation. This framework is not limited to COVID-19-related prevention and control. It also offers transferable guidance for educational institutions to implement routine emergency management when confronted with emerging infectious diseases and future public-health emergencies.

## 1 Introduction

Chinese government proposed that “In the face of the COVID-19 Pandemic, we insist on the supremacy of the people and life, insist on the dynamic clearance without wavering, and carry out the people's war, the overall war and the interdiction war against the pandemic.” Since the outbreak of COVID-19, most universities across the country have adopted a closed management and reduced mobility program in their campus policies based on the requirements of the central and local governments. In this situation, the university still adopts the same control measures as when the pandemic situation was severe, while lacking flexible policies and practical actions that match the students' psychological recognition. These measures protected the students' physical health to the maximum extent but ignored their psychological health. It is not only detrimental to the orderly development of the pandemic management work of the university itself, but also has a negative impact on the social stability in the context of the normalization of pandemic prevention and control.

Chinese government emphasized that pandemic prevention and control is not just a medical and health issue, but an all-round effort. To explore pandemic prevention and control of universities, it is necessary to take into account the special social status and campus situation of universities. As an important institution of higher education in society, each university should no longer simply stick to security factors in the new situation, but also actively and flexibly adjust their own policies, gradually implement conditional open mobility policies, and actively respond to evolving social dynamics and the expectations of internal and external groups. Therefore, the issues of how to further optimize policies in the process of university pandemic prevention according to the reality to find the highest total social benefit, how to make the pandemic prevention policies more widely accepted and thus be carried out in an orderly and sustainable manner, and how to optimize the governance system and governance capacity of university pandemic prevention and control have become important issues in the post-pandemic era. Taking the political cost of university pandemic prevention and control as the research object, based on the discussion of its connotation and importance, this paper attempts to study the evolution and spread of the political cost of university pandemic prevention and control through the establishment of an system dynamics theoretical model, including the four links of “establishment of political cost payment matrix—analysis of evolutionary equilibrium—small signal feedback—numerical simulation optimization”, Then, the chain evolution transmission mechanism and **governance approach for managing the political costs associated with university pandemic prevention and control**. Moreover, this will be an important step in measuring the political cost in the pandemic governance system. This paper has important theoretical significance and practical value for improving the efficiency of government and university pandemic prevention and control, realize the coordinated development of the social economy and the public health, solving local crises and maintaining national political security and stability. it contributes to broadening the research perspective of pandemic governance.

To avoid conceptual ambiguity, we first operationalize three core concepts. Political cost refers to the loss of political resources, legitimacy and authority that a governing actor—here, the university—incurs when its prevention-and-control decisions provoke resistance, erode trust, or generate negative externalities. Its observable indicators include the degree of inconsistency between policy formulation and implementation, the intensity and scale of student negative behavioral responses (escalating from online voicing to offline mobilization), the erosion of institutional legitimacy and public trust, and the magnitude of compensation or remediation the actor must pay to defuse the situation; in the model it is captured by the parameter *C*_2_ (the political cost of government–university integration) and functions as a feedback signal for system stability. Value balance denotes the dynamic equilibrium among the heterogeneous and partially conflicting value claims of universities (order and performance goals), students (health and economic interests, psychological and identity needs) and media (influence and discourse power), such that no actor's core value is depressed below an acceptable threshold; its indicators include the relative intensity of students' economic vs. health appeals, the gap between official discourse and actual implementation, and students' value/psychological identification with the policy. Identity value (value identification) refers to students' psychological recognition of the legitimacy and fairness of the university's policy (corresponding to *E*_3_ and *L*_3_ in the model); when it is too low, it drives negative participation. The three are related as follows: extreme depletion of political cost is both a consequence and a driver of value imbalance—once students' value identification collapses, they shift from economic to health appeals and from rational individual negative behavioral responses to negative/collective negative behavioral responses, destabilizing the system.

Internationally, the effectiveness of public health crisis governance depends not only on policy content but, crucially, on the quality of interaction, trust and institutional resilience among multiple actors. Existing research shows that public health outcomes are shaped by the interaction of structural, institutional and contextual factors, in which governance quality and post-crisis dynamics are particularly critical, calling for integrated policy frameworks to manage public health emergencies (1–4). Recent regional evidence further establishes resilience as a cornerstone of primary health care and crisis governance (5), while endogenous and exogenous factors continue to shape population health outcomes in the post-pandemic period (6). Although the world has entered a post-pandemic phase, routinized emergency preparedness for emerging infectious diseases makes the question of how to preserve governance legitimacy and social acceptance while ensuring safety even more pressing in practice—precisely the tension highlighted by the university setting examined here.

Building on this background and gap, this study addresses three research questions. RQ1: How do the strategic interactions among universities, social media and students evolve under different political-cost conditions during a university public health emergency? RQ2: How does political cost, acting as a feedback signal, determine the stability of the tripartite governance system, and what are its critical thresholds? RQ3: How can the university pandemic-governance system be optimized along the system–process–value dimensions to achieve a stable and legitimate win–win equilibrium transferable to future public health emergencies? Accordingly, we propose three testable propositions. H1: University governance behavior is the dominant destabilizing factor across equilibria—under sustained student collective negative behavioral responses, the system becomes unstable whenever the university adopts passive governance, regardless of the media's strategy. H2: Political cost *C*_2_ is a feedback signal with critical thresholds—within a bounded range it sustains the optimal cooperative equilibrium, whereas extreme depletion drives students from economic to health appeals and into negative participation, destabilizing the system. H3: Optimization along the structure–process–value dimensions (flattening the emergency organization, whole-process participation channels, and value compensation) shifts the system toward a stable and legitimate equilibrium and generalizes to non-COVID public health emergencies.

This study makes three contributions. First, theoretically, it explicitly introduces political cost into a multi-actor evolutionary game of university public health emergencies and uncovers its mechanism as a feedback signal for system stability. Second, methodologically, it brings the small-signal model together with root-locus and participation-factor analysis into evolutionary game theory, making the marginal influence of each actor on stability quantitatively measurable. Third, in policy terms, it proposes a three-dimensional system–process–value optimization framework that yields an operational and transferable pathway for governance improvement.

## 2 Literature review

### 2.1 Current status of internal and external governance system of Chinese universities

#### 2.1.1 Changes in the external governance system of universities

In the context of normalization of pandemic prevention and control, the orderly implementation of pandemic prevention and control in universities is an important link to avoid concentrated outbreaks. Due to unique historical and institutional reasons, the traditional university governance system in China has been dominated by the government for a long time, and its governance model has certain peculiarities (15). In recent years, China has started to try to combine government governance and corporate governance to build a modern university system with Chinese characteristics, and establish a sound governance model led by the party committee, managed by the president and participated by teachers and students (18). It tries to introduce consultative democracy system in the innovation of university management system to adjust various relationships between internal and external universities and build university governance system (17). Besides, realizing the differences in roles, status, roles, demands, values and interests between different governance subjects in universities and society, it takes the initiative to build a social governance community (20). It strives to break the blocking factors of information disclosure from the government, society, universities and individuals at multiple levels in order to balance information resources and external situation, balance administrative power and academic autonomy, empower the improvement of university governance capacity and realize the modernization of university governance capacity (19).

#### 2.1.2 Internal governance system adjustment of universities

The outbreak of the Covid-19 pandemic has posed a significant challenge to university governance.. It has been found that in the context of normalization of pandemic prevention and control, the orderly implementation of pandemic prevention and control in universities is an important part of avoiding concentrated outbreaks, while universities themselves are at the core of a stereoscopic cross-shaped net-like governance system, which is multiple participation from universities, government departments, students, media, and social organizations (11, 12, 14). Specifically, universities accept the leadership of several departments such as the Ministry of Education, the municipal government, and the municipal education commission, integrate and implement the relevant policies of each department and makes relevant recommendations, and formulate a public health master plan for the hospital that is compatible with its own development to fully guarantee the life safety and physical health of relevant personnel (13, 21); They also need to establish a sound long-term organizational guarantee that can ensure the smooth flow of governmental orders for the university. The public health and pandemic prevention system should be established to ensure that the relevant pandemic prevention and control policies are put into practice (10, 11). At the same time, actively accepting the treatment and feedback of relevant opinions, strengthening health supervision and controlling risks in key areas, and creating a harmonious and healthy teaching environment are also important (16). In addition, Universities served as the main force in infectious disease prevention and control among medical and nursing staff and the role of the main position of science popularization, ensure the continuity of teaching and research activities, and provide precise health education and ideological education to university students with knowledge gaps, panic and anxiety (13). Finally, they give full play to their own initiative in accordance with the social and even international environment, and seek support for the smooth implementation of pandemic prevention work and reduce the risk of pandemics (11, 21).

### 2.2 Review of the current status of research

For China the road of building a strong socialist modern state has been opened, and the governance of universities has been given a new connotation. Therefore, it is necessary to analyze the elements and sort out the relationship of university governance process and create a more effective governance system based on the changing times and the development needs of higher education. The outbreak of the COVID-19 pandemic has produced new challenges and new opportunities for university governance. Traditional research on university governance is fragmented, usually evaluating or improving one or several aspects, and lacks the attempt to comprehensively promote the modernization of university governance capacity and establish a dynamic governance mechanism centered on universities themselves by systematically analyzing and sorting out the internal logic and multiple interests of university governance, forming a multi-governance governance system.

### 2.3 International literature, theoretical integration, and the research gap

Existing research on university governance tends to focus on one or a few facets, is dominated by domestic experience, and rarely engages with international public health crisis governance or systematically integrates the internal logic and plural interests of the governance process. Internationally, the very core of crisis governance is the interdependence and systemic feedback among actors: health-system policy processes are shaped by the roles, relationships and power dynamics of multiple actors (3); governance capacity and institutional quality are key determinants of public health outcomes (1); and structural and contextual factors (e.g., economic uncertainty, environmental exposure) affect health outputs through institutional mediation, underscoring the need for integrated policy frameworks (2, 4); in the post-crisis phase, system resilience becomes the cornerstone for sustaining essential services and governance legitimacy (5, 6).

Theoretically, the present modeling implicitly draws on three frameworks that should be made explicit. First, institutional theory: universities are embedded in a tiao-kuai (vertical–horizontal) environment with multiple lines of authority, whose rigid rules and path dependence explain why one-size-fits-all measures persist even after conditions improve. Second, public choice theory: governments and universities, face transaction costs in supplying prevention-and-control public goods and cannot simultaneously accommodate the claims of the media and other stakeholders, thereby entering a continuous game. Third, behavioral economics and bounded rationality: students and media are not fully rational, and their strategy adjustment follows replicator-dynamics-style learning and imitation, with risk perception, fairness judgments and psychological identification jointly driving participation choices.

This identifies a clear research gap: existing studies lack a unified analytical framework that simultaneously captures (i) multi-actor strategic interaction, (ii) the feedback mechanism of political cost, and (iii) each actor's marginal contribution to system stability. This study fills the gap with a political-cost-driven tripartite evolutionary game combined with small-signal measurement, and maps the literature onto the model one-to-one: institutional theory corresponds to universities' active/passive governance strategies and constraints (*C*_1_, *C*_3_, α); public choice theory corresponds to political cost and the punishment–reward mechanism (*C*_2_, *P*, A); and behavioral economics corresponds to students' psychological gains/losses and negative behavioral responses risk (*E*_3_, *L*_3_, β).

## 3 Case introduction

### 3.1 Case design and data sources

This section adopts an illustrative single-case design for case analysis. Universities in City-T was selected as a critical case through information-oriented sampling (7). As one of the major Chinese cities that experienced a large-scale Omicron outbreak under the dynamic-zero-COVID policy in early 2022, the publicly documented university-student interactions in this city allow the complete tripartite governance dynamics to be clearly observed within a compressed time window under severe political-cost pressure, which delivers high reference value for the governance mechanism investigated in this research.

The case narrative is reconstructed via triangulation based on three categories of publicly available materials: (i) Official policy documents: pandemic-management directives issued by the Education Commission of City-T and university-level implementation documents; (ii) Media coverage; (iii) Student interviews.

This case serves primarily for illustration rather than hypothesis testing. It aims to identify various behavioral strategies that constitute the strategy space of the evolutionary-game model elaborated in the subsequent sections, including proactive governance versus passive governance, objective media guidance versus distorted media guidance, and individual negative behavioral responses versus collective negative behavioral responses. It also empirically validates the plausibility of the escalation chain formalized by the model. The mapping between case dynamics and game variables is presented in the final paragraph of this section. This case does not constitute an independent empirical test for the model predictions. All outputs of the model are conclusions derived from theoretical deduction. Systematic empirical tests using independently collected governance-effect data are discussed as a supplementary research agenda in Section 6.2.

### 3.2 Pandemic situation and governance measures

After the detection of the Omicron variant in City-T in January 2022, schools across the city entered an early winter break and implemented strict closed-campus management. In March, universities actively prepared for the return of students. Although the pandemic situation in City-T had improved relatively, sporadic new infections still occurred. Considering that returning students came from all parts of the country, universities maintained online teaching at the initial stage of student resumption. As the pandemic further improved and community-level COVID-19 infections were brought under control, universities gradually resumed offline teaching.

On April 2022, University-A formally shifted from full closed-campus management to “strict campus control”. Offline teaching was restored, faculty members were allowed to commute normally, while student mobility remained restricted, with exceptions granted only for students meeting specific requirements. Teaching and living areas of the campus were accessible to teachers and part of campus service staff, whereas student access to campus premises was subject to strict restrictions. Interviews indicate that the above-mentioned arrangements triggered psychological imbalance among students. Despite multiple higher-education work directives released by the Education Commission of City-T in the dynamic-zero-COVID phase, many universities failed to take practical actions to dynamically adjust their policies. Student discontent kept accumulating, and students from some universities voiced their grievances on various online media platforms. Universities noticed such public opinions but failed to fully recognize the psychological factors driving students' public expressions of demands. Later, under timely governmental guidance, universities adjusted their campus pandemic-prevention policies. They carried out teaching and research activities in an orderly manner while safeguarding the health of campus stakeholders and achieved important progress in pandemic response.

### 3.3 Analytical generalization

While the universities in City-T case offers an in-depth illustration of the political-cost mechanism, structurally comparable governance dynamics were independently observed in universities of two other cities during the 2022 Omicron wave, which strengthens the external plausibility of the core logic of this model. At University-B, Ma et al. (8) reported that depressive symptoms among quarantined students surged from 9.1 % to 36.1 % within two weeks of dormitory-based confinement. This finding confirms that the psychological pathway from restrictive governance to student distress—the driver of political-cost accumulation in our model (Section 4)—exists beyond the context of City-T.

At University-C in City-H, Yuan et al. (14) documented a parallel evolutionary trend by analyzing 32-month online-forum datasets: students evolved from initial compliance to growing impatience, and eventually developed negative behavioral responses toward campus self-policing, especially during the second Omicron wave. This observation aligns with the passive-governance strategy captured by the political-cost parameter *C*_2_ in our model. Conducted in different cities, the two studies, one adopting mental-health surveys and the other adopting online-forum keyword analysis, corroborate distinct components of the same underlying mechanism: the psychological-cost pathway (8) and the governance-structure pattern (14).

Noticeable contextual differences among the three cities reinforce, rather than weaken, the argument for analytical generalizability (7). Three dimensions of variation are particularly informative.

First, governance tensions manifested in different institutional forms. In universities in City-T, tensions stemmed from *tiao-kuai* misalignment: gaps between municipal-government policy directives and discretionary implementation by universities. Universities in City-S operated under city-wide centralized lockdown, exercising institutional discretion within overarching municipal mandates. By contrast, University-C functioned as a single-institution self-policing system: campus closure was enforced by its own administrative bodies, without the inter-institutional coordination tensions observed in the City-T and City-S cases (14).

Second, student negative behavioral responses varied in form and intensity. Negative behavioral responses among City-T students originated from accumulated psychological imbalance; student negative behavioral responses in City-S were triggered by specific incidents; negative behavioral responses at University-C in City-H were largely confined to online platforms, demonstrating that the underlying mechanism does not depend on reaching a particular mobilization threshold.

Third, and most theoretically significant, the policy-justification contexts differed considerably across sites. Universities in City-T maintained closed-campus measures even after community-level COVID-19 elimination. City-H kept campuses under strict self-policing as a spillover-prevention measure despite zero local infections amid the City-S outbreak (14). Universities in City-S implemented campus lockdowns at the peak of the Omicron wave, when public-health rationales appeared most compelling. Nevertheless, student discontent emerged in all three contexts. This carries important theoretical implications: the political-cost mechanism governing strategic interdependence among universities, students and media depends less on the objective public-health rationality of policies, and more on stakeholder perceptions of fairness and transparency in institutional implementation.

These cross-city similarities and contrasts show that the tripartite governance pattern identified in this paper is not unique to City-T. The identical escalation chain emerges across diverse governance structures, triggers for negative behavioral responses and policy-justification contexts, and its underlying psychological mechanism receives independent corroboration from at least one other city (8). Therefore, this mechanism possesses cross-contextual validity: it is activated whenever restrictive campus governance accumulates political costs among governed stakeholders, whether under *tiao-kuai* fragmented governance, centralized municipal mandates, or single-institution self-regulation. Accordingly, this case is treated as an analytically selected representative of a broader class of governance dynamics, in which political cost mediates strategic interdependence among institutional authority, public discourse and collective action (7). Systematic multi-city comparative case study is prioritized as a future-research direction in Section 6.2 and lies outside the scope of this paper. Instead, analytical generalization is achieved through two embedded university subunits providing mutually corroborating within-case evidence.

In the post-pandemic era, COVID-19 prevention has transitioned from an emergent public-health event to a routine public-health concern. Correspondingly, policy management should become less urgent and less exclusionary. From the perspective of public-choice theory, governmental bodies and academic institutions, as rational economic actors and institutional agents, are obligated to deliver essential public goods related to pandemic governance for students. Throughout governance processes, these actors inevitably incur a set of “transaction costs”, limiting their capacity to reconcile conflicting claims from social-media platforms and other stakeholders. Conversely, diverse stakeholders shape pandemic-response policies through multiple channels, which ultimately drives these policies toward equilibrium. Since pandemic governance involves numerous stakeholders and frequent interest conflicts alongside substantial externalities, actors engage in continuous games to safeguard their respective interests. This hinders comprehensive and effective policy implementation and undermines the overall performance of pandemic prevention and control.

In the above-mentioned case, supervision, altruism and reciprocity characterize the interactions among the three game actors: universities, social media and students, which is consistent with evolutionary-game theory (see Figure 1). The fundamental assumptions underpinning this model are as follows. All three actors in the game exhibit core traits of the homo-economicus assumption: they consistently weigh costs against benefits and seek to maximise their own payoffs. In addition, universities, as rational actors, adjust their behavioral strategies according to their own preferences in the course of game evolution.

**Figure 1:**
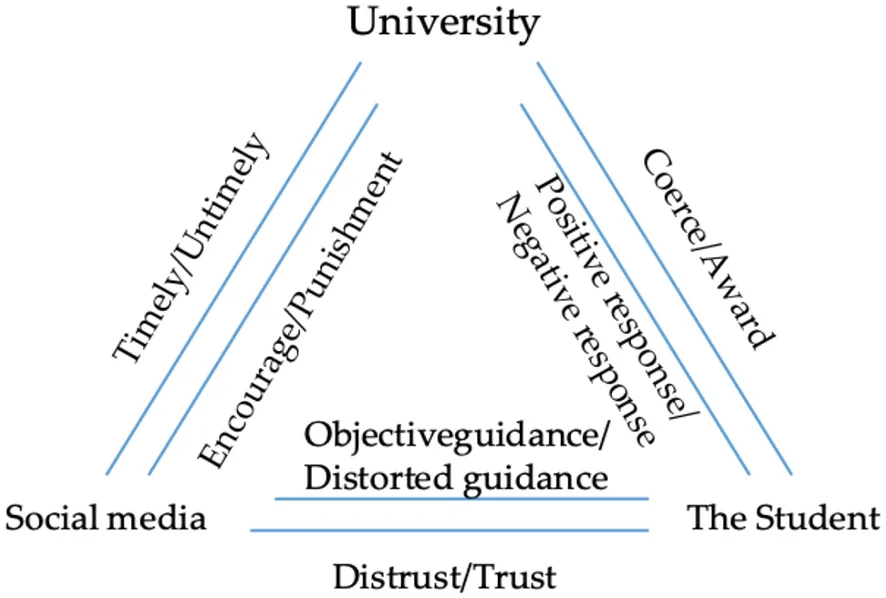
A model of university-social media-student dynamics in pandemic governance.

Case dynamics directly map onto the three game variables. Universities' persistence in closed-campus management after contextual improvements and their tendency toward delay when confronted with student appeals correspond to their passive-governance strategy and the accumulated political cost *C*_2_. The shift of student appeals from online voicing to offline expression reflects their strategic transition from individual (rational) negative behavioral responses to collective negative behavioural responses, alongside the substitution of health-centered demands for economic-centered demands. Media choices between official narratives and student claims correspond to its objective- versus distorted-guidance strategies. The chain of events uncovered by this case is exactly what the subsequent model formalizes and subjects to stability analysis.

## 4 Evolutionary game modeling and equilibrium analysis

This study uses a systems-modeling approach to characterize the multi-actor strategic interactions in university public health emergency governance. Specifically, evolutionary game theory is used to represent the strategy choices of universities, social media and students; under the bounded-rationality assumption, a payoff matrix is used for fitness analysis, the single-population evolutionarily stable strategy is obtained by solving the replicator dynamics equations, and these are combined into the three-dimensional dynamical system (*K*); the asymptotic stability of the eight pure-strategy equilibria is determined from the signs of the Jacobian eigenvalues (Lyapunov criterion); a small-signal model then linearizes the system, with the root locus characterizing the migration of the characteristic roots and the stability thresholds as the key parameter (political cost *C*_2_) varies, and participation factors measuring the relative contribution of the three state variables—the probabilities of student collective negative behavioral responses *x*, media objective guidance *y*, and active university governance *z*—to each modal component, thereby identifying the dominant governance actor at each equilibrium. The numerical simulation is implemented in Python with state vector Δ*X* = [*x, y, z*]^*T*^; a corresponding parameter set is specified for each equilibrium (Tables 1, 2; Appendices A, B), and convergence is verified using random initial values.

**Table 1:** Parameter values of all variables at balanced point *I*_1_.

Variable	Parameter value	Variable	Parameter value	Variable	Parameter value	Variable	Parameter value
*C*_1_	1	α	0.5	*C*_4_	1	*C*_5_	1
*C*_2_	1	*E*_1_	1	*R*_3_	2	β	0.7
*C*_3_	2	*L*_1_	2	*R*_4_	5	*E*_3_	3
*R*_1_	5	*A*	2	*E*_2_	1	*L*_3_	1
*R*_2_	10	*P*	3	*L*_2_	0.5	*T*	5

**Table 2:** Parameter values of all variables at balanced point *I*_4_.

Variable	Parameter value	Variable	Parameter value	Variable	Parameter value	Variable	Parameter value
*C*_1_	1	α	0.5	*C*_4_	1	*C*_5_	3
*C*_2_	4	*E*_1_	1	*R*_3_	2	β	0.7
*C*_3_	2	*L*_1_	2	*R*_4_	3	*E*_3_	1
*R*_1_	5	*A*	2	*E*_2_	1	*L*_3_	1
*R*_2_	5	*P*	3	*L*_2_	0.5	*T*	1

### 4.1 Evolutionary game modeling

It should be emphasized that the rational-economic-man assumption here is a tractable baseline rather than a complete description of behavior. First, the model uses replicator dynamics rather than fully rational best responses, and thus essentially characterizes how boundedly rational actors gradually adjust their strategies and converge toward equilibrium through learning, imitation and trial and error, consistent with behavioral economics. Second, institutional and psychological dimensions are not excluded but endogenized through the payoff parameters: universities' constraints and regulatory capacity enter via α and *C*_3_; students' psychological gains and losses and fairness perceptions enter via *E*_3_, *L*_3_ and β; and political cost *C*_2_ precisely compresses sociopolitical dimensions—legitimacy, trust and social acceptance—into an analyzable feedback signal. The rational-economic-man assumption is therefore a modeling starting point into which institutional and behavioral factors are incorporated in parameterized form. Introducing richer psychological and institutional heterogeneity (e.g., risk attitudes under prospect theory, group identity and emotional contagion) is a valuable extension.

Within the context of pandemic governance, universities, social media platforms, and students engage in dynamic interactions that shape the evolutionary trajectory of the game. Grounded in the rational economic man assumption, each type of actor bases its strategic choices on the pursuit of self-interest maximization. Consequently, a payoff matrix is employed to conduct fitness analysis for the participating entities, after which the system's single-population evolutionary stable strategy (ESS) is derived by solving the replicator dynamics Equation 1. Grounded in the rational economic man assumption, students serve as both primary participants and interest-bearing subjects in pandemic governance. Comprising a heterogeneous collection of autonomous individuals, the student body inherently exhibits diverse demands, as well as varying perceptions and acceptance thresholds concerning disease transmission (7). Nevertheless, students universally express the same requirements for a good health. In addition, the student has the same economic needs. The student will have different strategic options in the evolutionary game. The response of the student is based on their perception of risk. In terms of cost, major pandemics have obvious negative external characteristics, which will damage or reduce the student's physical and mental health and fixed assets to a large extent. In terms of earnings, pandemic prevention and control will have huge long-term earnings, its premise is based on the student, where soon return to the benefits of economic growth, solving the employment problem and so on, but it can be seen that the negative externalities brought by borne by the student, and make the student in terms of cost—benefit imbalance, as a rational economic man, The cost-benefit imbalance is bound to cause student discontent and negative behavioral responses. This means that in the process of epidemic preparedness and response, the government needs to pay corresponding compensation measures to the student. Therefore, the student will have two behavioral strategies: “collective negative behavioral responses” and “individual negative behavioral responses”.

The student collective negative behavioral responses shows as follows: (1) Negative anti pandemic behaviors, such as damage to public facilities and public goods. (2) Negative willingness to fight the pandemic, such as using some media, contacting a wider range of the public and even all kinds of people in society to carry out a larger scale of anti-pandemic actions. (3) Negative demand for compensation, such as “asking the government or social organizations” for their own pandemic prevention and control compensation, asking for more subsidies and social security subsidies, etc. The performance of the student in individual negative behavioral responses is as follows: (1) the student actively understands the relevant situation and knowledge through the formal media channels or websites, and makes reasonable and reasonable comments or voice in the media. (2) Actively communicate with relevant departments or express interest demands of pandemic prevention through official channels of the government, and exercise their rights and interests through lawful means, such as government online forums, etc. (3) Reasonably and legally participate in pandemic governance to safeguard their rights and interests.

1. Social media, under the rational economic man hypothesis, serves as a key actor in public relations. Social media is a collective organization with common activities established by the university and the student in order to achieve certain goals and follow certain policies, systems and service purposes, including social organizations, media and industry associations. With the nature of social groups, social media pursue their own interests, such as organizational benefits, social influence and discourse power. Media information platforms (Weibo, WeChat official account, Tik-Tok official account, etc.) provide a buffer zone for the student to negotiate and negotiate with the university even government on “pandemic prevention” affairs—the “public domain” of pandemic prevention and control. Therefore, there are two behavioral strategies of social media in pandemic prevention and control: “objective guidance” and “distorted guidance”.

The distorted guidance of social media is shown as follows: (1) Some social media are forced to tangle with the government, and at the same time are limited by the news management system. In the context of public health emergencies, there may be a collective “Silence” phenomenon. (2) Under the temptation of vicious competition in the industry and driven by other interests, many we media distorted the reports on some “pandemic prevention” events. (3) In order to chase click-through rates or gain public attention, social-media platforms disseminated or disclosed certain pandemic-prevention-related incidents, which aroused negative emotions such as panic among the public. The objective guidance of social media is as follows: (1) In the early stage of pandemic prevention and control, actively collect public opinion, supervise and distort reports, and timely feedback to relevant departments such as the government. (2) During the outbreak period, assist the government and other relevant departments to do propaganda work, actively popularize relevant knowledge to the public, and actively participate in the government pandemic assessment, prevention and Control Symposium and demonstration meeting, and make positive reports. (3) In the late stage of pandemic prevention and control, we should actively assist the government in releasing official information, actively pacify the public's unstable mood, and do a good job of “bridge” between the public and the government.

2. In pandemic prevention and control, the university has the following preferences: First, as an “agent”, it will bind its own interests with those of the “principal”, the student. The government entrusts universities with the pursuit of national strategic objectives, including the attainment of socioeconomic value, the fulfillment of ideological aspirations, and the maintenance of a dominant position in pandemic governance. In fulfilling this mandate, universities are obliged to serve the public interest of the broader society. The government, in turn, steers the processes and behaviors related to student participation. They actively provide the student with timely and good anti-pandemic public goods and services, and take the public interest into account when making pandemic prevention and control decisions. At the same time, the current performance appraisal of the government in the aspect of “anti-pandemic” and stability maintenance is still a “one-vote-power” system, so the government can't help choosing positive governance strategies in the pandemic prevention and control, and it may have an impact on the university. Second, as the main governing body, the university will also pursue the maximization of its own interests in daily operation like the government, reflecting the hypothesis of rational economic man. For example, some university governance mechanism and mode are still subject to “tournament”, negative handling public negative behavioral responses and so on, very accord with the preference of university decision-making. Additionally, the university's effectiveness is hampered by constraints related to monitoring technology, supervisory practices, and other institutional limitations. Therefore, the university often produces two behavioral strategies in pandemic prevention: “positive governance” and “negative governance”.

The university's negative governance is as follows: (1) in the early or middle stage of the outbreak, the university conceals information about the pandemic, or controls the media, resulting in information asymmetry of students. (2) The university does not take into account the student interest, lacks a sound communication mechanism, feedback mechanism and public appeal expression mechanism, and lacks cost awareness. (3) Indifferent to the appeal of student interest, more behavior or benefit compensation to their own and can bring benefits to their own departments. (4) The university is constrained by its technological capabilities, supervisory methods, and other institutional limitations. The university's positive governance is reflected in the following: (1) The information is released timely and widely. (2) The university and the student can communicate well in all aspects, so that students' opinions and interest demands can be better expressed, and can make decisions with reference to the opinions of all parties. (3) The university is in accordance with the law in all aspects of “pandemic prevention”, and can well help the government supervise and deploy all departments and institutions such as enterprises, social media and social organizations. (4) In the later stage, it can give reasonable compensation to the student and accept supervision of student.

Based on the above analysis, the variables and revenue matrix of the student, social media and the university under their respective behavioral strategies can be obtained, as shown in Tables 3, 4.

**Table 3:** Variables and definitions.

Variables	Definitions	Variables	Definitions
*C*_1_	The university's cost of “fighting the pandemic”, mainly including blocked expenditure, welfare compensation and other preferential policies	*R*_3_	The benefits of social media after objectively guiding pandemic
*C*_2_	The political cost of government and university integration	*R*_4_	The benefits of social media misrepresenting anti-pandemic practices
*C*_3_	The university's cost of pandemic control, namely the cost and loss when the government forces to deal with the incident or delays the negotiation, as well as the cost of information disclosure and possible liabilities	*L*_1_	The damage to the university caused by social media's misrepresentation of the pandemic
*C*_4_	The cost of the social media involvement in pandemic governance	*L*_2_	The reputational loss from the student when social media distorts guidance
*R*_1_	The revenue earned by the university's efforts to “fight the pandemic”	*C_5_*	The opportunity cost of student engagement in pandemic governance
*R*_2_	The benefits of the university's passive response to the pandemic	*E*_1_	The benefit that social media brings to the university when objectively guiding pandemic
α	This indicates the university's passive pandemic governance intensity	*E*_2_	The reputational benefits obtained from the student during the objective guidance of social media
β	The risk factor for individual student negative behavioral responses	*E*_3_	The psychological benefit that the student gets from the university's efforts to “fight the pandemic”
*T*	The university's compensation for student negative behavioral responses when it actively “fights the pandemic”	*L*_3_	The psychological loss of the student from the university's passive “fight against the pandemic”
*P*	The university's punishment for distorting social media to guide the fight against the pandemic by government	*A*	The university's reward to social media for objectively leading the fight against the pandemic by government

**Table 4:** University (government agent)–social media–student payoff matrix.

Game object				Government	
				Positive governance *z*	Negative governance 1 – *z*
**The public**	**Collective negative behavioral responses** ***x***	**Social media**	**Objective guidance** ***y***	−*C*5 + *T* + *E*3 −*C*4 + *A* + *R*3 + *E*2 −*C*1 + *C*2 − *C*3 + *R*1 + *E*1 − *A* − *T*	−−*C*_5_ −−*L*_3_ −−*C*_4_ +*R*_3_ +*E*_2_ −−α*C*_2_ −−*C*_3_ +*R*_2_ +*E*_1_
			Distorted guidance 1 – *y*	−−*C*_5_ +*T*+*E*_3_ −−*C*_4_ +*R*_4_ −−*L*_2_ –* P* −−*C*_1_ +*C*_2_ −−*C*_3_ +*R*_1_ −−*L*_1_ *+ P – T*	−−*C*_5_ −−*L*_3_ −−*C*_4_ +*R*_4_ −−*L*_2_ –* αP* −−α*C*_2_ −−*C*_3_ +*R*_2_ +α*P*−−*L*_1_
	Individual negative behavioral responses 1 – *x*	Social media	Objective guidance *y*	−−β*C*_5_ +β*T*+β*E*_3_ −−*C*_4_ +*A*+*R*_3_ +β*E*_2_ −−*C*_1_ +*C*_2_ −−β*C*_3_ +*R*_1_ +*E*_1_ –* A – βT*	−−β*C*_5_ −−β*L*_3_ −−*C*_4_ +*R*_3_ +β*E*_2_ −−α*C*_2_ −−β*C*_3_ +*R*_2_ +*E*_1_
			Distorted guidance 1 – *y*	−−β*C*_5_ +β*T*+β*E*_3_ −−*C*_4_ +*R*_4_ −−β*L*_2_ –* P* −−*C*_1_ +*C*_2_ −−β*C*_3_ +*R*_1_ −−*L*_1_ *+ P – βT*	−−β*C*_5_ −−β*L*_3_ −−*C*_4_ +*R*_4_ −−β*L*_2_ –* αP* −−α*C*_2_ −−β*C*_3_ +*R*_2_ +α*P*−−*L*_1_

At the heart of evolutionary game theory lies the concept of evolutionary stable strategy (ESS). ESS seeks to understand the principles by which players autonomously choose and adapt their strategies among alternative behavioral options. In the context of complex and stochastic interactions, this adaptive process propels the system toward an ESS, thereby giving rise to a stable macroscopic equilibrium. When in the evolutionary stable strategy, even if some individuals in the whole group change their strategies, this strategy change will interfere with the stability of the whole evolutionary game system for a short period, however, the presence of a particular strategy whose fitness exceeds the population average ensures that, following a phase of dynamic adjustment, those individuals that deviated will revert to their original strategies. Consequently, the game system ultimately returns to an evolutionary stable strategy (ESS) state.

In this paper, it is assumed that the probability of the student choosing a collective negative behavioral responses is *x*(0 ≤ *x* ≤ 1). Therefore, the probability of choosing an individual negative behavioral responses is 1 – *x*. At the same time, the probability of social media choosing objective guidance is *y*(0 ≤ *y* ≤ 1), and the probability of social media choosing distorted guidance is 1 – *y*. In addition, the probability that the university chooses the positive treatment is *z*(0 ≤ *z* ≤ 1), so the probability that the government chooses the negative treatment is 1 – *z*.

Expected benefits and population utility of collective and individual student choice negative behavioral responses are shown below:


$$\begin{array}{c} \begin{array}{c} U_{x1}=yz\left(-C_{5}+T+E_{3}\right)+y\left(1-z\right)\left(-C_{5}-L_{3}\right)\\ -\left(1-y\right)z\left(-C_{5}+T+E_{3}\right)+\left(1-y\right)\left(1-z\right)\left(-C_{5}-L_{3}\right)\\ U_{x2}=yz\left(-\beta C_{5}+\beta T+\beta E_{3}\right)+y\left(1-z\right)\left(-\beta C_{5}-\beta L_{3}\right)\\ +\left(1-y\right)z\left(-\beta C_{5}+\beta T+\beta E_{3}\right)+\left(1-y\right)\left(1-z\right)\left(-\beta C_{5}-\beta L_{3}\right)\\ {\bar{U}}_{x}=xU_{x1}+\left(1-x\right)U_{x2} \end{array} \end{array}$$ (1)

The average expected payoff of the student population is given by Equation 2:


$$\begin{array}{c} \begin{array}{c} U_{y1}=xz\left(-C_{4}+A+R_{3}+E_{2}\right)+x\left(1-z\right)\left(-C_{4}+R_{3}+E_{2}\right)\\ +\left(1-x\right)z\left(-C_{4}+A+R_{3}+\beta E_{2}\right)+\left(1-x\right)\left(1-z\right)\\ \left(-C_{4}+R_{3}+\beta E_{2}\right)\\ U_{y2}=xz\left(-C_{4}+R_{4}-L_{2}-P\right)+x\left(1-z\right)\\ \left(-C_{4}+R_{4}-L_{2}-\alpha P\right)+\left(1-x\right)z\left(-C_{4}+R_{4}-\beta L_{2}-P\right)\\ +\left(1-x\right)\left(1-z\right)\left(-C_{4}+R_{4}-\beta L_{2}-\alpha P\right)\\ {\bar{U}}_{y}=yU_{y1}+\left(1-y\right)U_{y2} \end{array} \end{array}$$ (2)

Expected benefits and population utility of university's choice of positive and negative treatment are shown below (Equation 3):


$$\begin{array}{c} \begin{array}{c} U_{z1}=xy\left(-C_{1}+C_{2}-C_{3}+R_{1}+E_{1}-A-T\right)\\ +x\left(1-y\right)\left(-C_{1}+C_{2}-C_{3}+R_{1}-L_{1}+P-T\right)\\ +\left(1-x\right)y\left(-C_{1}+C_{2}-\beta C_{3}+R_{1}+E_{1}-A-\beta T\right) \end{array}\\ \begin{array}{c} +\left(1-x\right)\left(1-y\right)\left(-C_{1}+C_{2}-\beta C_{3}+R_{1}-L_{1}+P-\beta T\right)\\ U_{z2}=xy\left(-\alpha C_{2}-C_{3}+R_{2}+E_{1}\right)\\ +x\left(1-y\right)\left(-\alpha C_{2}-C_{3}+R_{2}+\alpha P-L_{1}\right)\\ +\left(1-x\right)y\left(-\alpha C_{2}-\beta C_{3}+R_{2}+E_{1}\right)\\ +\left(1-x\right)\left(1-y\right)\left(-\alpha C_{2}-\beta C_{3}+R_{2}+\alpha P-L_{1}\right)\\ {\overset{¯}{U}}_{z}=zU_{z1}+\left(1-z\right)U_{z2} \end{array} \end{array}$$ (3)

Given that stakeholders—including universities, social media, and students—operate under conditions of incomplete information and that strategy shifts entail additional costs, these actors can only engage in boundedly rational strategic interactions. Drawing on the Malthusian principle, the replication dynamic equation serves as a suitable tool for analyzing information asymmetry in pandemic-related mass incidents through evolutionary game theory. This dynamic models the trajectory by which boundedly rational individuals adjust their strategies and converge toward equilibrium. Its underlying logic is that the frequency of a given strategy within a population shifts according to the gap between its expected payoff and the population's average payoff. Strategies yielding above-average returns are better adapted to the evolutionary process and more resilient against mutant strategies, leading to their increased adoption. Conversely, those yielding below-average returns are progressively abandoned. Ultimately, strategies with above-average payoffs prevail, while inferior ones are phased out. Formally, the replication dynamic equation expresses *dx*/*dt*—the growth rate of the strategy whereby the public opts for collective negative behavioral responses—as proportional to the difference between the payoff from that strategy and the average population payoff, as shown below (Equation 4):


$$\begin{array}{c} \frac{dx}{dt}=x\left(U_{x1}-{\bar{U}}_{x}\right)=x\left(1-x\right)\left(a+bz\right) \end{array}$$ (4)

Following the same logic, a replication dynamic equation can be established for the game between universities and social media. Combining the replication dynamic equations for all three stakeholder groups yields the three-dimensional dynamical system *(K)* presented below:


$$\{\begin{array}{c} F(x)=\frac{dx}{dt}=x(U_{x1}-{\bar{U}}_{x})=x(1-x)(a+bz)\\ F(y)=\frac{dy}{dt}=y(U_{y1}-{\bar{U}}_{y})=y(1-y)(c+dx+ez)\\ F(z)=\frac{dz}{dt}=z(U_{z1}-{\bar{U}}_{z})=z(1-z)(f+gx-ey) \end{array}\;$$ (5)

In order to simplify the operation, the intermediate variables from the above equation are defined as follows:


$$\begin{array}{c} \begin{array}{cc} a & =\left(1-\beta \right)\left(-C_{5}-L_{3}\right)\\ b & =\left(1-\beta \right)\left(T+E_{3}+L_{3}\right)\\ c & =R_{3}-R_{4}+\beta E_{2}+\beta L_{2}+\alpha P\\ d & =\left(1-\beta \right)\left(E_{2}+L_{2}\right)\\ e & =A+P-\alpha P\\ f & =-C_{1}+C_{2}+\alpha C_{2}+R_{1}-R_{2}-\beta T+\left(1-\alpha \right)P\\ g & =\left(\beta -1\right)T \end{array} \end{array}$$ (6)

### 4.2 Equilibrium analysis

Equilibrium analysis of the foregoing evolutionary dynamics yields the equilibrium points corresponding to four distinct cases:

The system admits eight pure-strategy equilibria involving all three populations, specifically: (0, 0, 0), (0, 0, 1), (0, 1, 0), (0, 1, 1), (1, 0, 0), (1, 0, 1), (1, 1, 0), and (1, 1, 1). Within system *(K)*, whenever *x, y*, and *z* assume binary values (0 or 1), the following equalities hold in the limit: $\frac{dx}{dt}=0,\;\frac{dy}{dt}=0,\;\frac{dz}{dt}=0$. It follows that all eight pure-strategy points listed previously are equilibrium points.

2. There are no duplicate populations adopting pure strategy equilibrium points:

$$\begin{array}{c} \left[\begin{array}{c} A:x=0,y=0,z\neq 0/1,F\left(x\right)=0,F\left(y\right)=0,F\left(z\right)\\ B:x=0,y=1,z\neq 0/1,F\left(x\right)=0,F\left(y\right)=0,F\left(z\right)\\ C:x=1,y=0,z\neq 0/1,F\left(x\right)=0,F\left(y\right)=0,F\left(z\right)\\ D:x=1,y=1,z\neq 0/1,F\left(x\right)=0,F\left(y\right)=0,F\left(z\right)\\ E:x=0,z=0,y\neq 0/1,F\left(x\right)=0,F\left(z\right)=0,F\left(y\right)\\ F:x=0,z=1,y\neq 0/1,F\left(x\right)=0,F\left(z\right)=0,F\left(y\right)\\ G:x=1,z=0,y\neq 0/1,F\left(x\right)=0,F\left(z\right)=0,F\left(y\right)\\ H:x=1,z=1,y\neq 0/1,F\left(x\right)=0,F\left(z\right)=0,F\left(y\right)\\ I:y=0,z=0,x\neq 0/1,F\left(y\right)=0,F\left(z\right)=0,F\left(x\right)\\ J:y=0,z=1,x\neq 0/1,F\left(y\right)=0,F\left(z\right)=0,F\left(x\right)\\ K:y=1,z=0,x\neq 0/1,F\left(y\right)=0,F\left(z\right)=0,F\left(x\right)\\ L:y=1,z=1,x\neq 0/1,F\left(y\right)=0,F\left(z\right)=0,F\left(x\right) \end{array}\right]\\ \;\Rightarrow Equationwithoutsolution \end{array}$$

3. Four single populations adopted pure-strategy equilibrium points as follows:

A: *When x* = 0 *and y, z*≠0 *or* 1,

$$\left\{\begin{array}{c} F(x)=0\\ F(y)=y(1-y)(c+ez)=0\\ F(z)=z(1-z)(f-ey)=0 \end{array}\;\Rightarrow (0,\frac{f}{e},-\frac{c}{e}\right)$$

B: *When x* = 1 *and y, z*≠0 *or* 1,

$$\left\{\begin{array}{c} F(x)=0\\ F(y)=y(1-y)(c+d+ez)=0\\ F(z)=z(1-z)(f+g-ey)=0 \end{array}\{\;\Rightarrow (1,\frac{f+g}{e},-\frac{c+d}{e}\right)$$

C: *When y* = 0 *and x, z*≠0 *or* 1,

$$\left\{\begin{array}{c} F(x)=x(1-x)(a+bz)=0\\ F(y)=0\\ F(z)=z(1-z)(f+gx)=0 \end{array}\;\Rightarrow (-\frac{f}{g},0,-\frac{a}{b}\right)$$

D: *When y* = 1 *and x, z*≠0 *or* 1,

$$\{\begin{array}{c} F(x)=x(1-x)(a+bz)=0\\ F(y)=0\\ F(z)=z(1-z)(f+gx-e)=0 \end{array}\;\Rightarrow (\frac{e-f}{g},1,-\frac{a}{b})\;$$

E: *When z* = 0 *and x, y*≠0 *or* 1,

$$\{\begin{array}{c} F(x)=x(1-x)a=0\\ F(y)=y(1-y)(c+dx)=0\\ F(z)=0 \end{array}\;\Rightarrow Equation\;without\;solution\;$$

F: *When z* = 1 *and x, y*≠0 *or* 1,

$$\{\begin{array}{c} F(x)=x(1-x)(a+b)=0\\ F(y)=y(1-y)(c+dx+e)=0\\ F(z)=0 \end{array}$$

4. There was one hybrid strategy balanced point, which can be described as follows:

When *x, y, z* ≠ 0 or 1, the system (*K*) satisfies the following equation:


$$\{\begin{array}{c} F(x)=x(1-x)(a+bz)=0\\ F(y)=y(1-y)(c+dx+ez)=0\\ F(z)=z(1-z)(f+gx-ey)=0 \end{array}\;\Rightarrow \{\begin{array}{c} a+bz=0\\ c+dx+ez=0\\ f+gx-ey=0 \end{array}$$

From the above three-dimensional first-order equations, we can draw the following conclusions:


$$\begin{array}{cc} x^{*} & =-\frac{bc-ae}{bd}\\ y^{*} & =\frac{bdf-bcg+aeg}{bde}\\ z^{*} & =-\frac{a}{b} \end{array}$$

Within the framework of multi-population evolutionary game theory, an equilibrium point *I* is evolutionarily stable if and only if it represents a strict Nash equilibrium, which in turn implies a pure-strategy equilibrium. Stability assessment of the eight pure-strategy equilibria derived earlier proceeds via Lyapunov's stability criterion. Specifically, an equilibrium point is asymptotically stable when all eigenvalues λ of its associated Jacobian matrix possess negative real parts. Instability occurs either when all eigenvalues are positive or under mixed-sign conditions—namely, one negative eigenvalue accompanied by two positive ones, or one positive eigenvalue alongside two negative ones. Hence, the sign pattern of the Jacobian's eigenvalues determines the stability properties of the system's equilibria. More precisely, a necessary and sufficient condition for an equilibrium of the replicator dynamics to constitute an evolutionary stable strategy is that all eigenvalues of its Jacobian matrix have negative real parts. The Jacobian matrix for system *(K)* is therefore formulated as follows (6):


$$\begin{array}{c} \left[\begin{array}{ccc} \left(1-2x\right)\left[a+bz\right] & 0 & bx\left(1-x\right)\\ dy\left(1-y\right) & \left(1-2y\right)\left(c+dx+ez\right) & ey\left(1-y\right)\\ gz\left(1-z\right) & -ez\left(1-z\right)\&\left(1-2z\right)\left(f+gx-ey\right) \end{array}\right] \end{array}$$

According to formula (6), the eight pure-strategy equilibrium points and their eigenvalues of the system (*K*) are shown in Table 5.

**Table 5:** Equilibrium points and eigenvalues of the system (*K*).

Equilibrium point	Jacobian matrix	Eigenvalues	Asymptotic stability condition
*I*_1_(0, 0, 0)	*J*_1_=$\left|\;\begin{array}{ccc} a & 0 & 0\\ 0 & c & 0\\ 0 & 0 & f \end{array}\;\right|$	λ_1_ = *a*, λ_2_ = *c*, λ_3_ = *f*	λ_1_ <0 λ_2_ <0 λ_3_ <0
*I*_2_(0, 0, 1)	*J*_2_=$\left|\;\begin{array}{ccc} a+b & 0 & 0\\ 0 & c+e & 0\\ 0 & 0 & -f \end{array}\;\right|$	λ_1_=*a*+*b*, λ_2_=*c*+*e*, λ_3_=−*f*	λ_1_ <0 λ_2_ <0 λ_3_ <0
*I*_3_(0, 1, 0)	*J*_3_=$\left|\;\begin{array}{ccc} a & 0 & 0\\ 0 & -c & 0\\ 0 & 0 & f-e \end{array}\;\right|$	λ_1_=*a*, λ_2_=−*c*, λ_3_=*f*−*e*	λ_1_ <0 λ_2_ <0 λ_3_ <0
*I*_4_(0, 1, 1)	*J*_4_=$\left|\;\begin{array}{ccc} a+b & 0 & 0\\ 0 & -c-e & 0\\ 0 & 0 & e-f \end{array}\;\right|$	λ_1_=*a*+*b*, λ_2_=−*c*−*e*, λ_3_=*e*−*f*	λ_1_ <0 λ_2_ <0 λ_3_ <0
*I*_5_(1, 0, 0)	*J*_5_=$\left|\;\begin{array}{ccc} -a & 0 & 0\\ 0 & c+d & 0\\ 0 & 0 & f+g \end{array}\;\right|$	λ_1_=−*a*, λ_2_=*c*+*d*, λ_3_=*f*+*g*	Instability
*I*_6_(1, 0, 1)	*J*_6_=$\left|\;\begin{array}{ccc} -a-b & 0 & 0\\ 0 & c+d+e & 0\\ 0 & 0 & -f-g \end{array}\;\right|$	λ_1_=−*a*−*b*, λ_2_=*c*+*d*+*e*, λ_3_=−*f*−*g*	λ_1_ <0 λ_2_ <0 λ_3_ <0
*I*_7_(1, 1, 0)	*J*_7_=$\left|\;\begin{array}{ccc} -a & 0 & 0\\ 0 & -c-d & 0\\ 0 & 0 & f-e+g \end{array}\;\right|$	λ_1_=−*a*, λ_2_=−*c*−*d*, λ_3_=*f*−*e*+*g*	Instability
*I*_8_(1, 1, 1)	*J*_8_=$\left|\begin{array}{ccc} -a-b & 0 & 0\\ 0 & -c-d-e & 0\\ 0 & 0 & e-f-g \end{array}\right|$	λ_1_=−*a*−*b*, λ_2_=−*c*−*d*−*e*, λ_3_=*e*−*f*−*g*	λ_1_ <0 λ_2_ <0 λ_3_ <0

With reference to Table 5, the approach for determining the stability of each equilibrium point is illustrated using *I*_1_(0, 0, 0) and *I*_5_(1, 0, 0) as examples. At equilibrium point *I*_1_(0, 0, 0), the Jacobian matrix of system *(K)* is given by:


$$\begin{array}{c} J_{1}=|\;\begin{array}{ccc} a & 0 & 0\\ 0 & c & 0\\ 0 & 0 & f \end{array}\;| \end{array}$$

Under the above conditions, the eigenvalues of the Jacobian matrix are as follows: λ_1_ = *a*, λ_2_ = *c*, λ_3_ = *f* . When *a* < 0, *d* < 0 and *g* < 0, all eigenvalues are less than zero, so *I*_1_(0, 0, 0) is asymptotically stable, and this is termed the convergence point. When *a* – *b* – *c* > 0, *c* < 0, *f* < 0, all eigenvalues are greater than zero, so *I*_1_ (0, 0, 0) is unstable, and this is known as source. When the eigenvalue signs are mixed—specifically, one negative accompanied by two positives, or one positive alongside two negatives, *I*_1_(0, 0, 0) is unstable and it becomes a saddle point. Similarly, the asymptotic stability of *I*_2_, *I*_3_, *I*_4_, *I*_6_, *I*_8_ can be obtained.

At equilibrium point *I*_5_(1, 0, 0) the Jacobian matrix corresponding to system (*K*) is given by:


$$\begin{array}{c} J_{5}=|\begin{array}{ccc} -a & 0 & 0\\ 0 & c+d & 0\\ 0 & 0 & f+g \end{array}| \end{array}$$

At this point, the eigenvalue of Jacobian matrix is λ_1_ = −*a*, λ_2_ = *c*+*d*, λ_3_ = *f*+*g, since a* = (1−β)(−*C*_5_−*L*_3_) <0, λ_1_ = −*a*>0 does not meet the Lyapunov's stability condition, so *I*_5_ is an unstable equilibrium point. If λ_2_>0, λ_3_>0, then the I_5_ equilibrium point is the source; If λ_2_ <0 and λ_3_ <0, then *I*_5_ is the saddle point. From this we can get the asymptotic stability of point *I*_7_. *I*_5_(1, 0, 0) represents the collective student negative behavioral responses, social media distort guiding, the university negative processing in parallel. *I*_7_(1, 1, 0) represents the collective student negative behavioral responses objective guiding, the university, social media negative processing in parallel. In the reality of pandemic governance, these two situations of anarchy under the continuous collective student negative behavioral responses do not exist. As long as there is a major outbreak of pandemic events, the university will actively participate in the case of “stability maintenance” and “political cost”. Therefore, according to the stability of *I*_5_(1, 0, 0) and *I*_7_(1, 1, 0), when the student chooses the collective negative behavioral responses behavior strategy, system (*K*) will be unstable as long as the university chooses the negative behavior strategy, regardless of the distorted guidance of social media or objective guidance. Therefore, when the student chooses the behavior strategy of collective negative behavioral responses and the university chooses the negative treatment, the stability of the system has no relationship with the behavior strategy choice of social media, and the system is always unstable. The foregoing analysis indicates that, at this stage, the unstable points in the game involving universities, social media, and students are ill-defined, which aligns with real-world conditions. Accordingly, a simulation analysis was conducted for each equilibrium point as follows.

## 5 Development of a small-signal model and measurement simulation

While the equilibrium analysis above allows for qualitative judgments regarding the asymptotic stability of equilibrium points, it does not enable a precise description of how individual variables interact and influence system stability at each equilibrium point within system *(K)*. To address this limitation, a small-signal model is introduced to further scrutinize the stability of the eight equilibrium points. This approach is particularly pertinent to the measurement and analysis of political costs in evolutionary game theory.

### 5.1 . Small-signal modeling

A dynamical system's behavior—or its evolutionary trajectory—is characterized mathematically by a system of n-dimensional first-order non-linear ordinary differential equations. Here, n represents both the system's order and the count of its state variables. The system state itself is representable by any set of *n-*dimensional linearly independent variables associated with that state. From this, a minimal set of dynamical variables can be identified, enabling a complete description of both the system's behavior and its input variables. To construct a small-signal model, all non-linear state-space equations constituting the original system are linearized and recombined. The resulting expression is as follows:


$$\{\begin{array}{c} \frac{d\Delta X}{dt}=A\Delta X+B\Delta U\\ \Delta Y=C\Delta X+D\Delta U \end{array}$$ (7)

In the above Equation 7, Δ*X* is the *n*-dimensional state variable. Δ*Y* is the *m*-dimensional output variable. Δ*U* is the *r*-dimensional input variable. *A* is the matrix order *n* by *n*. *B* is the matrix order *n* by *r*. *C* is the matrix order *m* by *n*. and *D* is the matrices order *m* by *r*.

As can be seen from Equation 5 for the three-dimensional system, system (*K*) possesses relatively simple dynamic properties. By selecting appropriate state variables from the system and linearizing the non-linear state-space equations individually—in accordance with the small-signal model construction process described earlier—a corresponding small-signal model is obtained. This model further incorporates three-dimensional state variables and a 3 × 3 matrix A. The state variable **Δ*X*** is [*x, y, z*]^T^, and the state matrix ***A*** determine the Jacobian matrix (Equation 8), namely:


$$\begin{array}{c} \left[\begin{array}{ccc} \left(1-2x\right)\left[a+b\left(y-1\right)\right] & bx\left(1-x\right) & cx\left(1-x\right)\\ +c\left(z-1\right)\\ ey\left(1-y\right) & \left(1-2y\right)\left(d+ex-fz\right) & -fy\left(1-y\right)\\ 0 & fz\left(1-z\right) & \left(1-2z\right)\left(g+fy\right) \end{array}\right] \end{array}$$ (8)

### 5.2 . Stability analysis

From a principal-agent perspective, political cost encompasses both economic and sociopolitical dimensions. Without adequate oversight, universities may deviate from their agency duties to students during pandemic governance, triggering resistance and incurring political costs—a process characterized by ambiguity and incomplete-information signaling games. Universities must balance minimizing political expenditure against maximizing political returns. This dynamic fosters an evolutionary game among universities, social media, and students, wherein each party iteratively learns and adapts toward stable strategies. These strategic choices shape policy directions, pandemic control efforts, and student satisfaction, ultimately influencing political costs and system stability. The stability of political costs requires validation through evolutionary game testing. Thus, analyzing tripartite evolutionary interactions, establishing convergence conditions, and employing a small-signal model can enhance policy efficacy, achieve robust governance, and reduce negative externalities.

For effective system control, actual information must be promptly relayed to the governing body, which then adjusts its regulatory functions through organizational structures to ensure the achievement of control objective. By utilizing fuzzy system identification and functional simulation as methodological tools, and by taking system operating states, functions, and behaviors as units of analysis, complex system theory facilitates the stable control and equilibrium of dynamic systems amidst complex pandemic scenarios. This necessitates that the control subject be endowed with strong regulatory capacity. Political cost underscores the necessity for governments to strategically deploy political resources to stabilize and optimize the pandemic governance system within the framework of modern governance. The ultimate goal is to maximize political benefits while minimizing political costs. Therefore, the government needs to pay attention to reasonableness in terms of political costs, so as to ensure the stability of the governance system. When the system evolution game is at a non-ideal equilibrium point, by employing a small-signal model, it becomes possible to analyze parameter adjustments within a system and, ultimately, to achieve convergence to an ideal equilibrium point. Furthermore, measurement and analysis using this model allow for the effective derivation of optimal adjustment parameters that ensure rapid response under anti-interference conditions.

The aim of this paper is to offer an intuitive examination of how economic costs relate to political costs at various equilibrium points within pandemic governance. Using PYTHON, we implemented a simulation program based on the previously described small-signal model. Parameter sets were configured for each equilibrium point to ensure asymptotic system stability. Simulation analyses were then performed to explore the root locus and participation factors associated with different parameter choices. These analyses enable an evaluation of the impact of political cost on system stability and facilitate the identification of dominant governance actors at various equilibrium points. Due to space constraints, the analysis focuses on two representative points.

#### 5.2.1 Stability of I_1_

According to the initial value described above, the calculation shows that *I*_1_ satisfies *a* < 0, *c* < 0, *f* < 0. The game equilibrium analysis further indicates that, under the current conditions, equilibrium point *I*_1_ exhibits asymptotic stability. To assess the validity of the small-signal model, the initial values for *x, y*, and *z* were chosen at random. By taking *x* = 0.3, *y* = 0.6, *z* = 0.9 Using PYTHON, a dynamic evolution diagram for system *(K)* was constructed under the parameter values discussed above (see Figure 2). The diagram illustrates that, given the initial parameters, the steady-state values of *x, y* and *z* settle at [0, 0, 0]. This observation indicates that system (*K*) converges to equilibrium point *I*_1_ (0, 0, 0) under the chosen parameter configuration, thereby corroborating the small-signal model. Notably, the initial values of *x, y* and *z* were selected randomly; the system consistently evolves to the same final state regardless of the specific random assignment.

**Figure 2:**
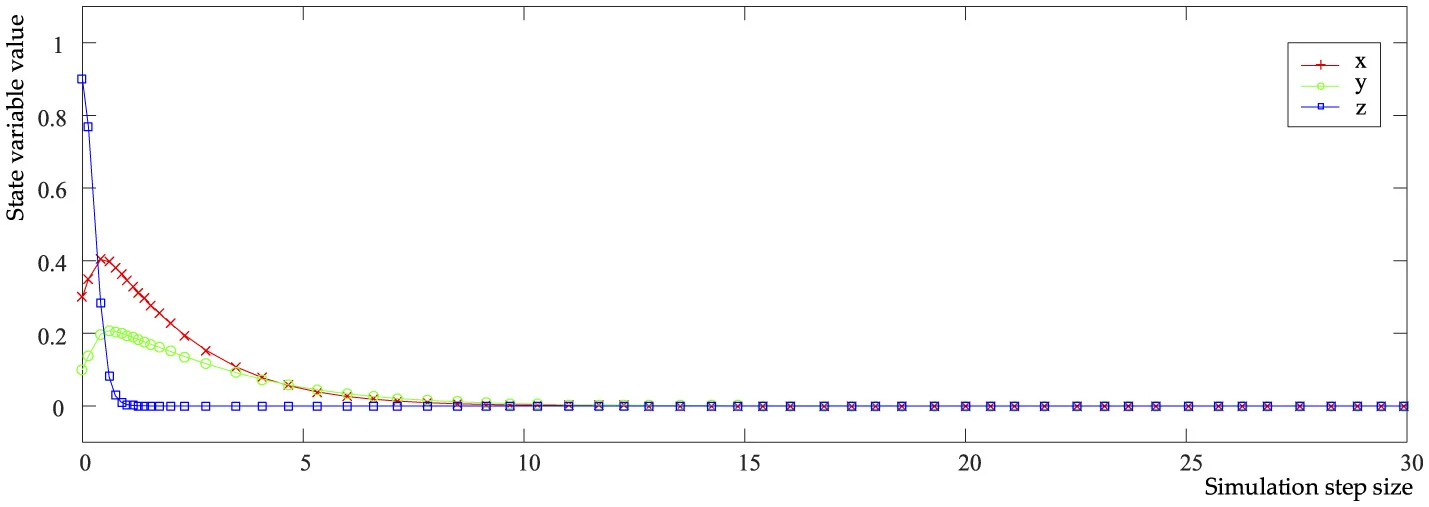
Dynamic evolution of system **(*K*)** at equilibrium point *I*_1_. The value of the state variable represents the value of **Δ*X***[*x, y, z*]^*T*^, where the probability of the public choosing a collective negative behavioral responses is *x*(0 ≤ *x* ≤ 1). At the same time, the probability of social media choosing objective guidance is *y*(0 ≤ *y* ≤ 1). In addition, the probability that the government chooses the positive treatment is *z*(0 ≤ *z* ≤ 1).

With the small-signal model validated, we next measured the political cost at equilibrium point *I*_1_. The parameter *C*_2_, representing government pandemic governance cost, was chosen to analyze system stability. Its impact on system *(K)* is examined as follows:

With the initial parameters from Table 1, *C*_2_ was increased from 0 to 10 in steps of 0.1. Figure 3a shows the corresponding root locus. As *C*_2_ rises, system (*K*) shifts from stable to unstable, with the stability threshold at *C*_2_ = 5.4. Hence, small-signal stability holds for *C*_2_ <5.4. Setting *C*_2_ = 5.4 while keeping other parameters constant confirms this result. In addition, if the value of *C*_2_ is 5.4, and other parameters are maintained, the conclusion can be verified. This shows that after the calculation*a* < 0, *c* < 0, *f* < 0. Equilibrium analysis of the game-theoretic model reveals that point I1 is a saddle point and thus unstable. Hence, the political cost *C*_2_ remains stable only within the interval [0, 5.4]. System stability declines progressively as *C*_2_ rises. Root locus analysis offers essential information on the trend, its determinants, and the stable parameter range that ensures overall system stability.

**Figure 3:**
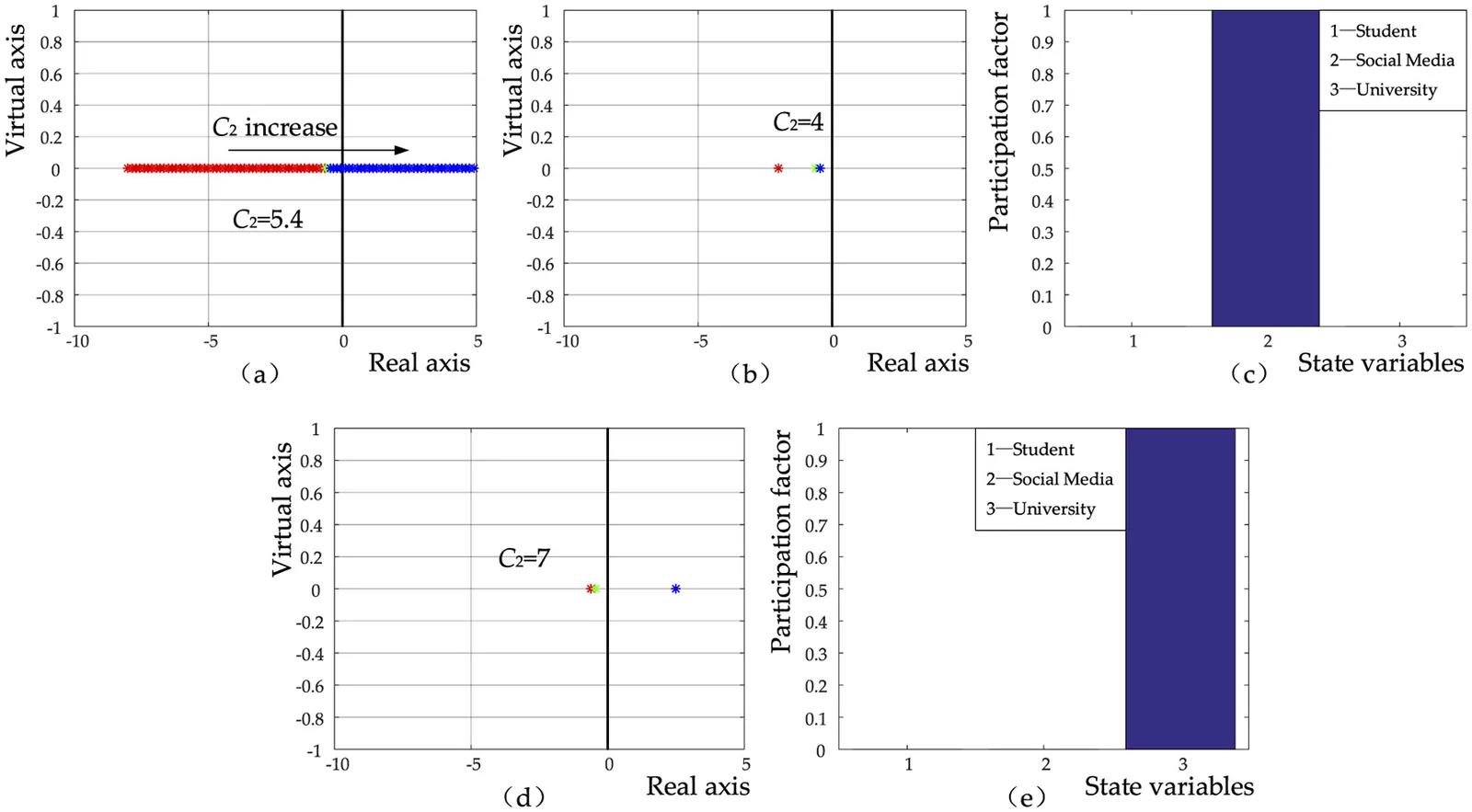
The influence of the political cost *C*_2_ of *I*_1_ on the stability of system **(*K*)**. **(a**, **b**, **d)** plot the root locus of system **(*K*)** when *C*_2_ takes different values (continuously varying or fixed), where the horizontal axis represents the real part of the system feature root, and the vertical axis represents the imaginary part of the system feature root. **(c**, **e)** plot the participation factors of the three state variables in system **(*K*)** when *C*_2_ takes different values.

Following the previous analysis, this study investigated the political cost under two distinct values: *C*_2_ = 4 and *C*_2_ = 7. These values correspond to stable and unstable system states, enabling an examination of participation by social media, government, and the public accordingly. The root locus for *C*_2_ = 4 is illustrated in Figure 3b. As shown, all three eigenvalues lie within the left half-plane, indicating stable system operation under this parameter setting. The most active participant in the game system is the social media, as shown in Figure 3c, which indicates that regulating social media behavior can play a decisive role in the stability of the system. Similarly, Figure 3d shows the root locus of the system when *C*_2_ = 7. The absence of all three eigenvalues from the left half-plane confirms the system's instability under this parameter setting. As presented in Figure 3e, the university emerges as the most active player in the game, implying that regulating university related behaviors is critical to system stability. The government emerges as the most active player in the game, implying that government behavior regulation is critical to system stability.

Stability analysis shows that when all three parties adopt negative strategies—distorted social media guidance, passive university governance, and individual student negative behavioral responses—the system's stability domain becomes highly sensitive to political cost *C*_2_. Here, *C*_2_ functions as a detrimental input; exceeding the threshold of 5.4 destabilizes the system, making it vulnerable to participant-induced disorder. Participation factor analysis reveals distinct dominant actors across system states: social media exerts the greatest influence during stability, while the university dominates in unstable conditions. Thus, at this equilibrium point, weaker social media distortion enhances system stability and resilience against interference.

#### 5.2.2 Stability of I_4_

Based on the initial value specified above, it is calculated that *I*_4_ satisfies *a* + *b* < 0, –*c* –*e* < 0, *e* – *f* < 0. Game equilibrium analysis further indicates that, given the current conditions, point I4 exhibits asymptotic stability. The small-signal model was subsequently tested by randomly selecting initial values for *x, y*, and *z*. By taking *x* = 0.3, *y* = 0.6, *z* = 0.9 as an example, this paper used PYTHON to create the dynamic evolution diagram of system (*K*) under the above parameter values, as shown in Figure 4. Under the given initial parameters, *x, y*, and *z* evolve to steady-state values of [0, 1, 1], indicating that system (*K*) settles at equilibrium point *I*_4_(0, 1, 1).

**Figure 4:**
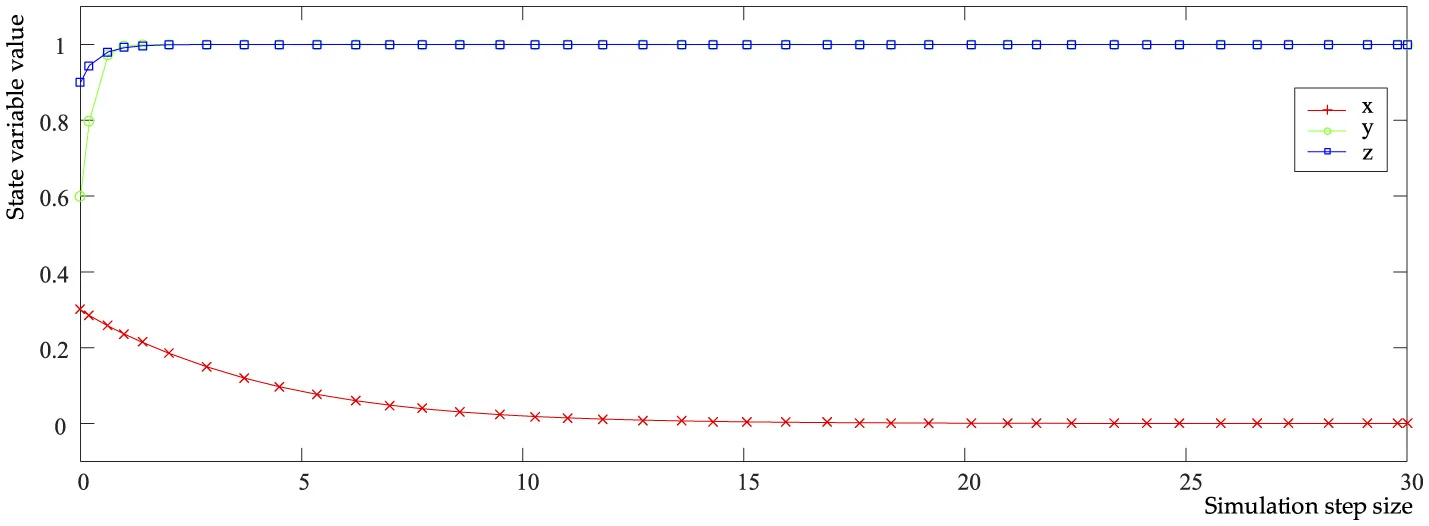
Dynamic evolution of system (*K*) at equilibrium point *I*_4_.

With the small-signal model confirmed to be accurate, a measurement and analysis of the political cost at the balanced point *I*_4_ was subsequently undertaken. Therefore, the political cost of governments' pandemic governance *C*_2_ was chosen as a parameter to analyze the stability of the system. The influence of the political cost *C*_2_ on the stability of system (*K*) was analyzed as follows:

With the initial parameters from Table 2, *C*_2_ was increased from 0 to 10 in steps of 0.1. Figure 5a shows the corresponding root locus. As *C*_2_ rises, system *(K)* shifts from unstable to stable, with the stability threshold at *C*_2_ = 2.4. Hence, small-signal stability holds for *C*_2_ > 2.4. Setting *C*_2_ = 2.4 while keeping other parameters constant confirms this result. This shows that after the calculation *a* + *b* < 0, –*c* –*e* < 0, *e* – *f* > 0. Equilibrium analysis of the game-theoretic model reveals that point *I*_4_ is an unstable saddle point. Hence, the political cost *C*_2_ maintains stability across the range [2.4, 10]. Moreover, higher values of *C*_2_ correspond to increased system stability.

**Figure 5:**
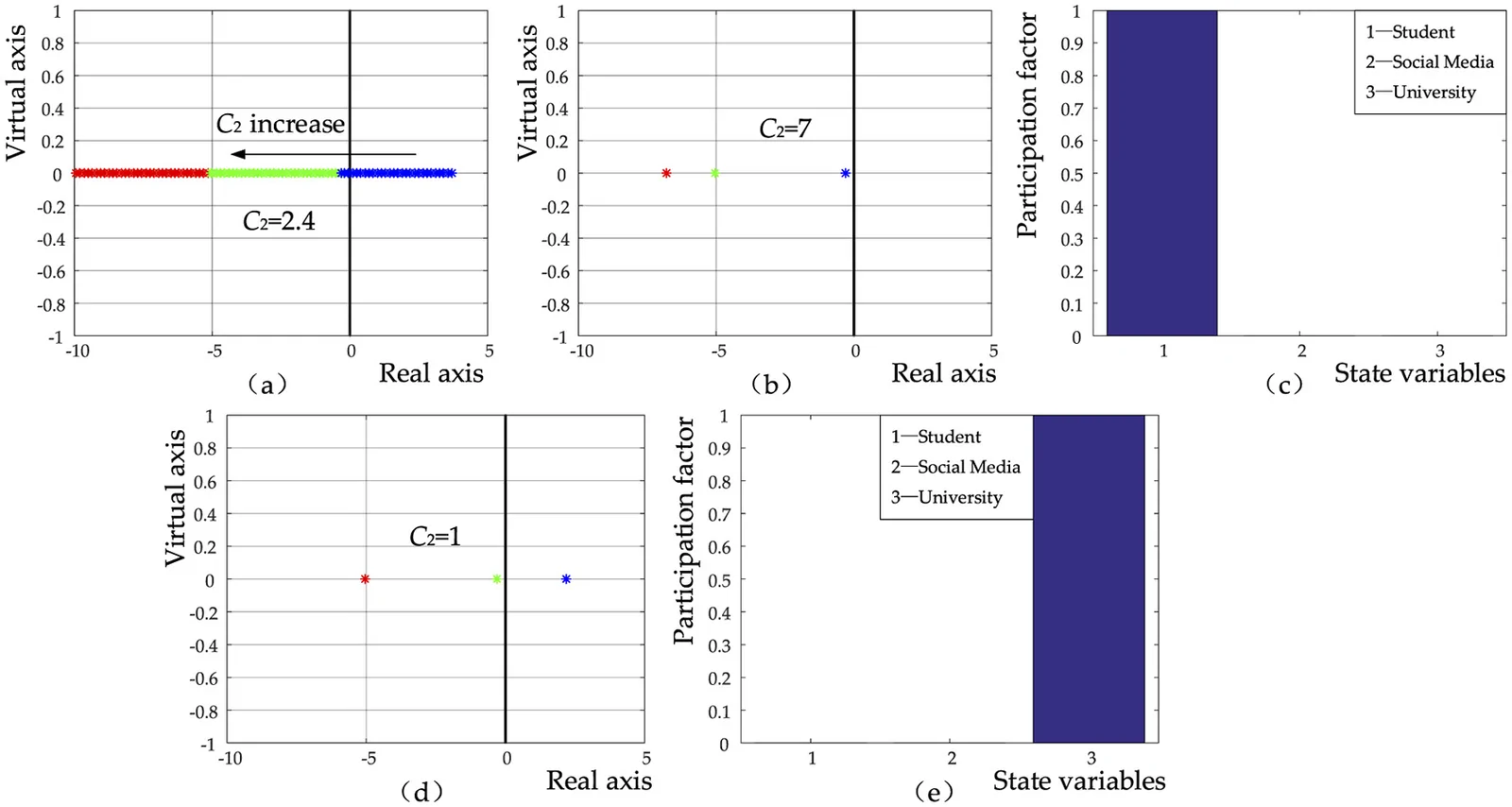
The influence of the political cost *C*_2_ of *I*_4_ on the stability of system (*K*). **(a)** Eigenvalue distribution on the complex plane for *C*_2_ = 2.4, the arrow indicates the direction of increasing *C*_2_; **(b)** Eigenvalue distribution on the complex plane for *C*_2_ = 7; **(c)** Participation factor bar chart for state variable 1 (Student); **(d)** Eigenvalue distribution on the complex plane for *C*_2_ = 1; **(e)** Participation factor bar chart for state variable 3 (University).

Following the analysis above, two political cost cases were examined, with *C*_2_ assigned values of 7 and 1, respectively. So that the participation of social media, universities, and the student can be analyzed when the system is stable and unstable. Figure 5b shows the root locus of the system when *C*_2_ = 7. The eigenvalues reside in the left half-plane for *C*_2_ = 7 (Figure 5c), confirming system stability, with the student as the most active participant—highlighting the critical role of student behavior regulation. In contrast, for *C*_2_ = 1 (Figure 5d), the eigenvalues lie outside the left half-plane, indicating instability. Under this condition, the university emerges as the dominant actor (Figure 5e), underscoring the decisive influence of university behavior on system stability.

The stability analysis above indicates that, during this phase, all three parties adopt positive behavioral strategies: objective guidance by social media, proactive governance by the university, and individual student negative behavioral responses. Under such conditions, the system's stability domain expands significantly in response to variations in the political cost *C*_2_. Within this strategic configuration, *C*_2_ exerts a positive influence on the system, representing benefits associated with political impact. Consequently, the findings demonstrate that the system maintains stability when *C*_2_ exceeds 2.4, and remains unaffected by participant interference under these circumstances.

Participation factor analysis further reveals distinct dominant actors across system states: students exert the greatest influence during stable conditions, whereas universities assume this role in unstable states. Thus, at this equilibrium point, weaker individual student negative behavioral responses enhance system stability and resilience against interference, while diminished proactive governance by universities exacerbates instability and reduces resistance to external disruptions. It can therefore be concluded that mitigating and guiding student negative behavioral responses behavior is key to preserving system stability at this equilibrium point.

In multi-party games, the payoff from proactive governance constitutes a critical determinant of university strategy. When universities adopt proactive strategies with low payoffs, rising political costs prompt a shift toward negative governance strategies vis-à-vis social media, triggering a transition from stability to instability. Conversely, when political costs are contained and the payoffs from proactive governance increase—derived from the participation of social media and students—universities become more inclined to maintain proactive strategies. This restores system stability, encourages rational behavior among students and social media, and curbs declines in their trust in universities. Conversely, low payoffs from proactive governance signal resistance from social media and insufficient student support, steering universities toward negative governance strategies. In this scenario, the evolutionary game among universities, social media, and students stabilizes around a strategy combination characterized by proactive governance, compliance, and active participation. This offers a viable solution to the information asymmetry problem in pandemic governance: prioritizing political costs, enhancing student participation, and increasing the payoffs associated with proactive university governance.

### 5.3 Sensitivity and scenario analysis

The stability measurement in this study itself constitutes a sensitivity and scenario analysis. On the one hand, a continuous single-parameter sweep of political cost *C*_2_ over [0, 10] in steps of 0.1 yields the root locus and stability thresholds (e.g., *C*_2_ = 5.4 at *I*_1_ and *C*_2_ = 2.4 at *I*_4_), characterizing the system's critical behavior as it shifts from stable to unstable (or vice versa)—a sensitivity characterization of the key parameter. On the other hand, the eight equilibria each correspond to a different strategy combination and parameter set (Tables 1, 2; Appendices A, B), amounting to a comparative analysis across eight governance scenarios; the switching of participation factors under different *C*_2_ values (e.g., *C*_2_ = 4 vs. *C*_2_ = 7) further reveals the migration of the dominant actor across scenarios. Taken together, the conclusions are robust to parameter variation: regardless of the specific values, as long as students persist in collective negative behavioral responses and the university governs passively, the system is invariably unstable (*I*_5_, *I*_7_); whereas when political cost is kept within a reasonable range and the university governs actively, the system tends toward the optimal equilibrium *I*_4_.

Further, the political-cost–student-psychology–participation-strategy mechanism chain revealed by the simulation has clear governance implications: when political cost is excessive depletion and value identification collapses, students' perception of health risk overrides economic considerations, and rational individual expression gives way to negative or even collective negative behavioral responses (β rises, *E*_3_ falls, *L*_3_ rises), so the system slides from stability to instability. This explains why purely rigid control crowds out the public's constructive participation, and suggests that the governance focus should shift from suppressing expression to sustaining identification through value compensation and psychological counseling.

## 6 Conclusions

### 6.1 Main conclusions and policy implications

Regarding the internal and external governance system of universities, American scholar Burton Clark believes that there are three systems of government power, academic authority and market coordination in the development of higher education, and it is through their coordinated interaction that the operation of higher education is possible. It is only in different times, different countries or different periods of the same country that the three forces combine with each other and the degree of their bias varies, thus forming different forms of higher education (9). This statement may not be comprehensive, but it reflects the importance of internal and external governance of universities in a pluralistic manner. Combining the above analysis results, we can find that the key problem to be solved in university epidemic governance is how to link different participating subjects through organizational arrangement and value identity, balance the interests of multiple subjects, and then optimize the university pandemic governance pattern and improve the effectiveness of university pandemic governance.

Integrating the stability, root-locus and participation-factor results across equilibria yields three clear conclusions. First, as Table 6 shows, the dominant participation factor in the instability domain of every equilibrium is the university (the government's agent), confirming H1: university governance behavior is the primary source of instability, and its strategy choice decisively affects system stability (the invariably unstable *I*_5_ and *I*_7_ being cases in point). Second, the threshold results for *C*_2_ (5.4 at *I*_1_, 2.4 at *I*_4_) confirm H2: political cost is a feedback signal with thresholds—at the optimal equilibrium *I*_4_, the system is stable when *C*_2_ > 2.4 and students' individual negative behavioral responses becomes the dominant factor in the stability domain, reflecting the dual role of a moderate political cost that both warns and empowers. Third, scenarios such as *I*_8_ (1, 1, 1) (Appendix B) show that when political cost is excessive depletion, students turn to negative/collective participation and the system destabilizes, confirming the value-imbalance-triggers-instability judgment in H3 and motivating the three-dimensional system–process–value optimization.

1. Grounded in the practical context of pandemic governance, this study establishes a foundational assumption that accounts for public and social media participation. Employing game equilibrium analysis, small-signal measurement, and numerical simulation, the research conducts a comprehensive equilibrium analysis of each equilibrium point, thereby identifying the evolutionary mechanisms governing political costs at these points. Through the foregoing analysis, the evolutionary game processes among the three governance actors are elucidated, alongside the measurement of their associated political costs. The results obtained for each equilibrium point throughout the game are summarized in Table 6. The findings reveal that the dominant participation factors responsible for maintaining system stability vary across equilibrium points. Notably, the analysis indicates that university governance behavior is the primary source of system instability, as evidenced by the leading participation factors observed at different stages. Consequently, within the evolutionary game framework that prioritizes political costs, the strategic choices of university governance behavior emerge as critically important, exerting a substantial influence on overall system stability. Furthermore, at the optimal equilibrium point *I*_4_, the student's individual negative behavioral responses behavior constitutes the dominant participation factor, playing a pivotal role in ensuring system stability.

**Table 6:** Distribution of dominant participation factors across equilibrium points in stable and unstable regions.

No.	Equilibrium point	Dominant participation factors in the stability domain	Dominant participation factors in the instability domain
1	*I*_1_(0, 0, 0)	Social media	University
2	*I*_2_(0, 0, 1)	Student	University
3	*I*_3_(0, 1, 0)	Student	University
4	*I*_4_(0, 1, 1)	Student	University
5	*I*_6_(1, 0, 1)	Student	University
6	*I*_8_(1, 1, 1)	Student	University

Meanwhile, Chinese universities generally adopt the organizational system of section hierarchy, which applies rigid and stable rules and systems. Although the emergency management system based on section hierarchy has the advantages of stable and authoritative order obedience mechanism and rapid response, it cannot independently identify and judge the environmental changes and their dangers, and does not pay attention to the anxiety and panic brought by “invisible risks”. When the unstable system dominant participant factor is the university, the responsibility of emergency response can be divested from the traditional organizational mechanism, and a flat organizational system can be built specifically to respond to emergencies, so as to promote the horizontal integration and vertical linkage of resources, strengthen the normality and relevance of policies, streamline policy content and implementation process, and This will enhance the efficiency of collaboration in governance and reduce the psychological stress of teachers and students.

2. At *I*_2_(0, 0, 1), the governance system enters a stagnation phase characterized by the onset of significant political cost losses (Appendix B). Here, the dominant participation factor in the stability domain shifts from social media to the student. Local governments and universities act as participants in both stable and unstable domains, as local governments shape policy implementation preferences based on regional economic benefits, taxes, and governance costs. Alignment between economic benefits and governance preferences motivates policy enforcement despite political costs; misalignment leads universities to incomplete implementation and responsibility avoidance. Given limited political resources, such behaviors escalate political cost consumption, undermining governmental authority and legitimacy. This negatively affects social stability, the business environment, policy execution, and local economic development. Countering the “ripple effect” of excessive political cost consumption during this stagnation phase requires an effective strategy from the unstable domain's dominant factor.

Thus, based on the process disconnects in university epidemic management derived from the above analysis, process reengineering should start from two aspects. First, many possible situational variables should be included in the consideration of process design to improve the feedback adjustment mechanism and avoid treating the epidemic with an isolated and static view. Make full use of and continue to develop existing information management platforms, and take the initiative to learn from governments, society and academia around the world to introduce advanced technologies from advanced experiences. Avoid disregarding changes in the situation and considering one end of the spectrum, which in turn makes it difficult to form consensus among policy actors and even invites public opinion crises. Second, focus on the construction of whole-process people's democracy. Be aware that process is intrinsic to democracy and that the people are the center of democracy building. In turn, we should expand the participation of different subjects in each stage of the epidemic management process, give them the opportunity to express their opinions, and include them in the consideration of policy formulation and implementation. Proactively build a “student- representative of student–teacher- representative of teacher-school-government” channel for the transmission of opinions, with lower-level executives regularly reporting daily information to higher-level executives and being monitored. The school should also liaise with the government and hold regular seminars with students, teachers and community representatives to provide a platform for direct communication between representatives of the various units of epidemic prevention and control.

3. At the optimal equilibrium *I*_4_, student behavior is the dominant factor maintaining stability and the core driver of political cost consumption. Student participation substitutes for local government efforts in pandemic remediation. Students should enhance health literacy and rationally defend their rights by engaging in governance and oversight under public rationality. For government, *I*_4_ signals both a warning against exploiting information asymmetry and an assurance to decisively manage pandemics, while fostering transparent collaboration with social media and enabling criticism from groups like students and media.

*I*_4_ generates positive tripartite effects: increased benefits from active governance reinforce universities' commitment, lowering political costs and stabilizing the system. However, it negatively impacts student mental health, necessitating internalization of pandemic governance externalities. This requires enhancing students' understanding to calibrate subsidies, strengthening psychological interventions, correcting biases, and promoting value recognition via official media and social organizations. Cross-organizational cooperation should broaden participation channels and reduce engagement costs.

4. In the evolutionary game, excessive depletion of political costs drives students toward negative participation and collective negative behavioral responses, destabilizing the system. *I*_8_(1, 1, 1) in local remediation shows that ignoring political costs at critical moments causes policy failure (Appendix B). Similarly, governments constrained by political costs risk failure through over-implementation. Higher rewards incentivize student participation, while stricter regulations increase negative responses. Pandemic governance thus creates a “crowding-out effect” on public engagement, reducing political costs and triggering instability.

According to the logic of incremental and interactive value formation, colleges and universities will be in contact with multiple parties in the society in the process of external governance, and the college prevention policy will also generate externalities to these organizations affected by the policy. In this process, universities should first actively establish good communication channels with social organizations, and make some exchanges between the costs paid and benefits obtained by both sides in the process of policy implementation, instead of treating themselves as an island of communication with the government only. At the same time, the school should work with the community to provide the material compensation and psychological counseling that students really need, and to consider the many details involved in the implementation process. For example, shopping vouchers are in most cases more effective than comfort items; pre-counseling is more difficult, but it can prevent problems from occurring instead of reducing them after they occur; only professional counseling can have a positive effect, and it is very easy for counselors to report information about students who go to counseling to counselors, which can cause students to resist counseling.

Building on these results, we propose operational policy recommendations along the three dimensions. Structural reorganization: (1) detach emergency response from the traditional bureaucracy and establish a dedicated, flattened emergency organization that integrates resources horizontally and links them vertically; (2) build a student–student-representative–teacher–teacher-representative–university–government channel for opinion transmission and oversight. Process reengineering: (3) incorporate situational variables into process design and establish feedback–adaptation mechanisms, avoiding an isolated, static view of the pandemic; (4) advance whole-process people's democracy by expanding the substantive participation of all actors at each stage of policy formulation and implementation, with regular forums for students, teachers and community representatives. Value distribution: (5) have the university and community jointly provide the material compensation students actually need (e.g., shopping vouchers are often more effective than comfort items) and psychological counseling, with interventions ideally front-loaded, delivered by professionals and kept strictly confidential; (6) use official media and social organizations to correct cognitive biases and enhance value identification, lowering participation costs and broadening participation channels to internalize the externalities of prevention and control.

Finally, it should be emphasized that the framework is not confined to COVID-19. Its object of analysis is governance dynamics rather than a specific pathogen: political cost as a feedback signal, value balance as a stability condition, and the system–process–value optimization apply to any public health emergency that triggers multi-actor interest conflicts and negative externalities. The study therefore in effect provides a disease-agnostic governance model for university public health emergencies: facing future emerging infectious diseases, public health disasters, or other campus emergencies, universities can use it to assess under which scenarios they become the dominant destabilizer and within which range the political cost lies, and can proactively sustain legitimacy and social acceptance through structural flattening, whole-process participation and value compensation. This is precisely the practical urgency of building routinized emergency-response capacity in the post-pandemic era.

### 6.2 Limitations and future research

Based on the system theory and cost-benefit theory, this article analyzes the epidemic prevention and control policies in City-T universities. On this basis, sorts out the value generated by epidemic prevention and control and considers its allocation methods, in response to the problems of insufficient students' right to information, large differences in governance policies of different groups, poor consistency between policy requirements and implementation, and neglect of students' mental health and reasonable demands in the current governance system of universities in the process of pandemic prevention and control. Based on the three-dimensional analysis framework of university epidemic governance, we propose the optimization plan and countermeasures for the university governance system from three aspects: structural reorganization, process reengineering and value distribution, so as to make it more scientific, realistic and vital.

To sum up, in the pandemic governance system, the relationships among the university, social media, and the student are constantly being reconstructed and developed interactively. In the post-epidemic era, universities should get rid of their over-reliance on government guidance and become active “helpers”; form a deeper awareness of their core position in the governance system of multiple subjects in universities, and continuously optimize the governance system to match it. In the face of major public health events, universities should form their own judgment on current affairs and pay attention to both real problems and potential crises. While giving full play to the ability of their own governance subjects, they should cooperate closely with other subjects, share the pressure and cost of each party in different situations, create and coordinate the distribution of benefits, so as to avoid both leniency and strictness, and further meet the realistic requirements of modernization of university governance system and governance ability.

First, the model builds on a rational-economic-man baseline. As discussed in Section 4.1, the replicator dynamics capture boundedly rational strategy adjustment through learning and imitation, providing a tractable starting point. Incorporating prospect-theoretic risk attitudes, group identity, and emotional contagion as explicit behavioral mechanisms would strengthen the model's psychological realism. Second, political cost *C*_2_ is treated as an exogenous parameter whose influence is mapped through sensitivity analysis rather than endogenously determined. Third, the case evidence in Section 3 serves an illustrative function—identifying behavioral strategies and confirming the empirical plausibility of the escalation chain—rather than constituting an independent empirical test of the model's predictions. Fourth, the empirical material is drawn from City-T universities under the Chinese *tiao-kuai* governance structure; although the cross-city comparison in Section 3 provides preliminary corroboration from City-S and City-H, systematic multi-case designs would be needed to establish the mechanism's generalizability across institutional contexts. Fifth, the system—process—value framework is developed within the COVID-19 context and remains at a conceptual level; its applicability to other public health emergencies requires further validation.

Future research could proceed along the following directions. First, the rational-economic-man baseline can be relaxed by introducing behavioral mechanisms such as prospect-theoretic risk attitudes, group identity and emotional contagion to develop a behaviorally more realistic extended model. Second, richer institutional heterogeneity (multiple lines of authority and vertical–horizontal relations) can be incorporated to characterize different governance contexts. Third, the system–process–value framework can be applied to other public health emergencies (e.g., emerging infectious diseases and mass public-health incidents) to test its applicable boundaries as a general emergency-governance model. More broadly, endogenizing political cost and subjecting the model's predictions to independent empirical testing represent two further directions for extending the present framework.

## Data Availability

The original contributions presented in the study are included in the article/Supplementary material, further inquiries can be directed to the corresponding author.
