# Supplementary material for: Political cost, value balance, and the optimization of university public health emergency governance: an evolutionary game and small signal analysis
Source: Front Public Health. 2026 Sep 16;14:1824985. doi: 10.3389/fpubh.2026.1824985 (PMC13625675; doi:10.3389/fpubh.2026.1824985)
Supplement: Supplementary file 1 [file Data_Sheet_1.docx]

**Appendix A**

Analyze *I*_3_ like *I*_1_, and see the results in Appendix A.

**Stability of *I*_3_**

**Table A-1.** Parameter values of all variables at balanced point *I_3_*

| **Variable** | **Parameter value** | **Variable** | **Parameter value** | **Variable** | **Parameter value** | **Variable** | **Parameter value** |
| --- | --- | --- | --- | --- | --- | --- | --- |
| *C*_1_ | 1 | *α* | 0.5 | *C*_4_ | 1 | *C*_5_ | 1 |
| *C*_2_ | 1 | *E*_1_ | 1 | *R*_3_ | 2 | *β* | 0.7 |
| *C*_3_ | 2 | *L*_1_ | 2 | *R*_4_ | 3 | *E*_3_ | 3 |
| *R*_1_ | 5 | *A* | 2 | *E*_2_ | 1 | *L*_3_ | 1 |
| *R*_2_ | 10 | *P* | 3 | *L*_2_ | 0.5 | *T* | 5 |

**Figure A-1.** Dynamic evolution of system (*K*) at equilibrium point *I_3_*

**Figure A-2.** The influence of the political cost *C*_2_ of *I*_3_ on the stability of system (*K*)

**Appendix B**

Analyze *I*_2_, *I*_6_, *I*_8_ like *I*_4_, and see the results in Appendix B

**1. Stability of *I*_2_**

**Table B-1.** Parameter values of all variables at balanced point *I_2_*

| **Variable** | **Parameter value** | **Variable** | **Parameter value** | **Variable** | **Parameter value** | **Variable** | **Parameter value** |
| --- | --- | --- | --- | --- | --- | --- | --- |
| *C*_1_ | 1 | *α* | 0.5 | *C*_4_ | 1 | *C*_5_ | 3 |
| *C*_2_ | 4 | *E*_1_ | 1 | *R*_3_ | 1 | *β* | 0.5 |
| *C*_3_ | 2 | *L*_1_ | 1 | *R*_4_ | 5 | *E*_3_ | 0.5 |
| *R*_1_ | 5 | *A* | 1 | *E*_2_ | 1 | *L*_3_ | 1 |
| *R*_2_ | 5 | *P* | 1 | *L*_2_ | 0.5 | *T* | 1 |

**Figure B-1.** Dynamic evolution of system (*K*) at equilibrium point *I_2_*

**Figure B-2.** The influence of the political cost *C*_2_ of *I*_2_ on the stability of system (*K*)

**2. Stability of *I*_6_**

**Table B-2.** Parameter values of all variables at balanced point *I_6_*

| **Variable** | **Parameter value** | **Variable** | **Parameter value** | **Variable** | **Parameter value** | **Variable** | **Parameter value** |
| --- | --- | --- | --- | --- | --- | --- | --- |
| *C*_1_ | 1 | *α* | 0.5 | *C*_4_ | 1 | *C*_5_ | 3 |
| *C*_2_ | 4 | *E*_1_ | 1 | *R*_3_ | 1 | *β* | 0.5 |
| *C*_3_ | 2 | *L*_1_ | 1 | *R*_4_ | 5 | *E*_3_ | 2 |
| *R*_1_ | 5 | *A* | 1 | *E*_2_ | 1 | *L*_3_ | 1 |
| *R*_2_ | 5 | *P* | 1 | *L*_2_ | 0.5 | *T* | 2 |

**Figure B-3.** Dynamic evolution of system (*K*) at equilibrium point *I_6_*

**Figure B-4.** The influence of the political cost *C*_2_ of *I*_6_ on the stability of system (*K*)

**3. Stability of *I*_8_**

**Table B-3.** Parameter values of all variables at balanced point *I_8_*

| **Variable** | **Parameter value** | **Variable** | **Parameter value** | **Variable** | **Parameter value** | **Variable** | **Parameter value** |
| --- | --- | --- | --- | --- | --- | --- | --- |
| *C*_1_ | 1 | *α* | 0.5 | *C*_4_ | 1 | *C*_5_ | 1 |
| *C*_2_ | 4 | *E*_1_ | 1 | *R*_3_ | 2 | *β* | 0.7 |
| *C*_3_ | 2 | *L*_1_ | 2 | *R*_4_ | 3 | *E*_3_ | 3 |
| *R*_1_ | 5 | *A* | 2 | *E*_2_ | 1 | *L*_3_ | 1 |
| *R*_2_ | 5 | *P* | 3 | *L*_2_ | 0.5 | *T* | 2 |

**Figure B-5.** Dynamic evolution of system (*K*) at equilibrium point *I_8_*

**Figure B-6.** The influence of the political cost *C*_2_ of *I*_8_ on the stability of system (*K*)
